# Harnessing artificial intelligence for enhanced veterinary diagnostics: A look to quality assurance, Part I Model development

**DOI:** 10.1111/vcp.13401

**Published:** 2024-12-05

**Authors:** Christina Pacholec, Bente Flatland, Hehuang Xie, Kurt Zimmerman

**Affiliations:** ^1^ Department of Biomedical Sciences and Pathobiology Virginia‐Maryland College of Veterinary Medicine Blacksburg Virginia USA; ^2^ Department of Biomedical and Diagnostic Sciences University of Tennessee Institute of Agriculture Knoxville Tennessee USA

## Abstract

Artificial intelligence (AI) has transformative potential in veterinary pathology in tasks ranging from cell enumeration and cancer detection to prognosis forecasting, virtual staining techniques, and individually tailored treatment plans. Preclinical testing and validation of AI systems (AIS) are critical to ensure diagnostic safety, efficacy, and dependability. In this two‐part series, challenges such as the AI chasm (ie, the discrepancy between the AIS model performance in research settings and real‐world applications) and ethical considerations (data privacy, algorithmic bias) are reviewed and underscore the importance of tailored quality assurance measures that address the nuances of AI in veterinary pathology. This review advocates for a multidisciplinary approach to AI development and implementation, focusing on image‐based tasks, highlighting the necessity for collaboration across veterinarians, computer scientists, and ethicists to successfully navigate the complex landscape of using AI in veterinary medicine. It calls for a concerted effort to bridge the AI chasm by addressing technical, ethical, and regulatory challenges, facilitating AI integration into veterinary pathology. The future of veterinary pathology must balance harnessing AI's potential while intentionally mitigating its risks, ensuring the welfare of animals and the integrity of the veterinary profession are safeguarded. Part I of this review focuses on considerations for model development, and Part II focuses on external validation of AI.

## 1 INTRODUCTION

### 1.1 The evolution of artificial intelligence (AI) in medicine

AI using computers to perform tasks or solve problems, has existed since the 1950s.^1^ Since its inception, AI has gone through periods of dormancy and bursts of advancements across many scientific fields. Most people use AI daily, even if they are unaware of it. Better‐known applications of AI include Siri (Apple Inc., Cupertino, CA, USA) and Alexa (Amazon, Seattle, WA, USA). The use of AI in medicine has been slower to evolve. Recent advancements, such as digitization of records, greater computational power, data storage at a more reasonable cost, and the development of deep learning models, have facilitated the expansion of AI into the medical field.^2^ In pathology, advances in whole slide scanners, including improved resolution, better magnification capabilities, shorter scanning times, affordable data storage, and lower cost, have aided the development of AI.^3^

### 1.2 AI applications in veterinary cytology and histopathology

In anatomic pathology, AI can count cells or mitotic figures, predict prognoses, and screen for, detect, and classify cancer.^4^, ^5^, ^6^ AI can also determine the tumor tissue origin in cancers of unknown histogenesis,^7^ determine genomic instability, and predict responses to therapy.^8^ In clinical pathology, AI can perform cell identification/classification and differential counts in hematopathology, evaluate poikilocytosis, and detect hemic neoplasia.^9^ Often, AI performs at least as well as experts in the respective specialty.^2^ Novel applications such as staining normalization,^10^, ^11^ virtual staining of unstained slides,^12^ and generation of synthetic data to allow model application to rare diseases (small dataset) that might not otherwise be amenable to study have all shown remarkable success.^13^ AI helps fill a needed niche within medicine for tests that are fast, reliable, objective, reusable (ie, applicable to more than one patient), and cost‐effective.^14^ Additionally, some aspects, such as virtual slide staining, could positively impact environmental sustainability within the field. This begs the question, why is AI not more widely used in veterinary diagnostic laboratory medicine?

The answer to this question is complex. Many papers discuss the issues that must be overcome to achieve widespread acceptance of AI in medicine, but few offer empirical evidence supporting how to solve them.^14^ An important concept, the “AI chasm,” refers to the discrepancy between the model performance of an AI in a research setting and real‐world application. The AI chasm describes how even if an AI has high model performance in the developmental environment, the AI may not be accurate or generalizable in real‐world diagnostic settings.^15^, ^16^, ^17^ Another significant limitation of AI moving into the medical field is the lack of standardized quality assurance for the validation and maintenance of AI. In traditional veterinary clinical pathology, clinical pathologists and medical technologists carry out new method validation, verification, and quality assurance techniques, teach these procedures to others, and ensure that labs adhere to analytical quality goals to the highest degree possible. To maintain high standards of accuracy and dependability in veterinary laboratory medicine, laboratory personnel must be equipped with the necessary knowledge and skills to develop and implement AI efficiently and confidently. By implementing unique quality assurance practices, we can ensure that reliable and high‐quality tests remain the norm in veterinary laboratory medicine.

Goals of this two‐part review are to (1) introduce basic AI terminology and (2) provide an understanding of the rigorous and unique preclinical evaluation AI must undergo before implementation in a clinical setting. An overview of AI development stages for image‐based tasks is presented, focusing on pre‐clinical development and evaluation of AI‐based decision support systems such as neural networks. While this manuscript uses examples of image‐based tasks, many of the same topics relate to text‐based tasks solved with other machine learning models (eg, support vector machines, k‐nearest neighbor, naïve Bayes). Significant differences in definitions between traditional diagnostic laboratory quality terminology and AI terminology are highlighted. The intended audience is veterinary pathologists and laboratory technologists involved in developing or implementing AI.

## 2 PRECLINICAL EVALUATION FOR AI SYSTEMS

**FIGURE 1:**
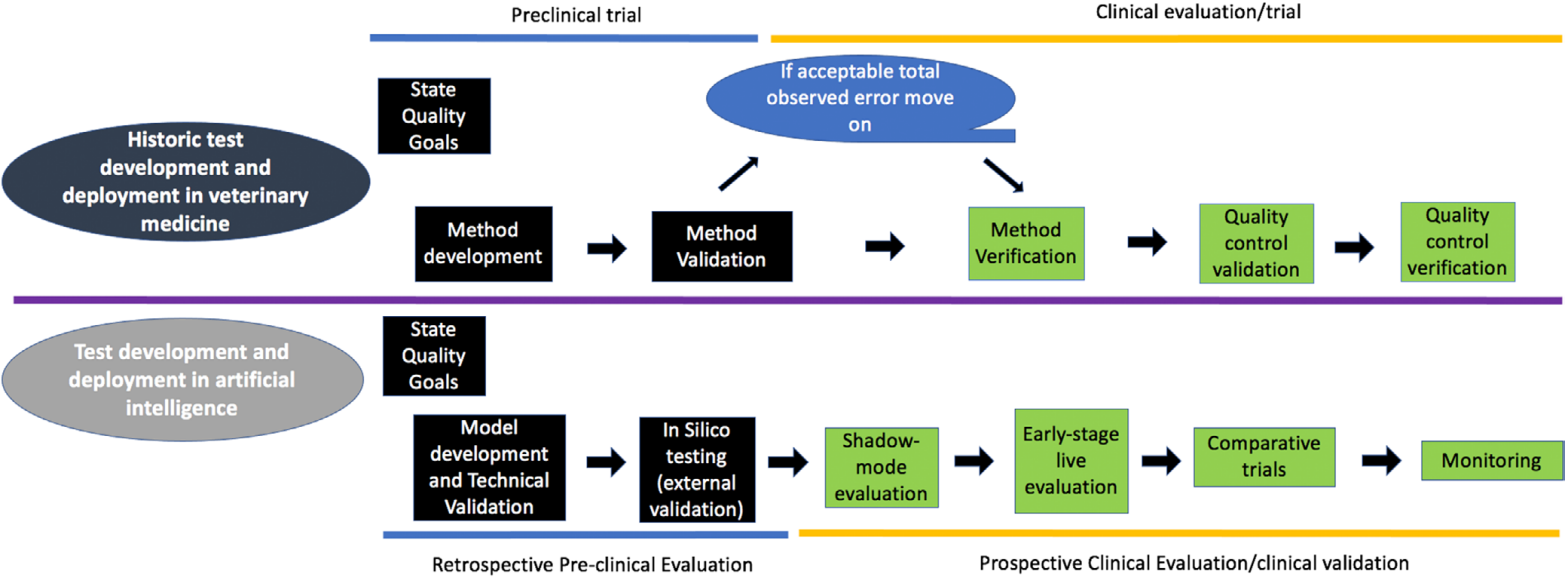
Schematic diagram comparing the stages of development for historic diagnostic tests (eg, hematology and chemistry analyzers) in veterinary medicine and artificial intelligence. The diagram divides the stages into preclinical and clinical evaluation of a new test. The stages of development and deployment in the schematic for veterinary medicine are those recommended by the American Society for Veterinary Clinical Pathology (ASVCP) Quality Assurance and Laboratory Standards (QALS) Guidelines.^48^ The stages of development and deployment in the schematic for artificial intelligence, are adapted from Figure 1 and Table 1 of the Developmental and Exploratory Clinical Investigations of Decision support systems driving by Artificial Intelligence (DECIDE‐AI) guidelines^19^ and Figure 2 of the DECIDE‐AI: A new reporting guideline and its relevance to artificial intelligence studies in radiology paper.^20^

AI validation requires a unique approach differing from test development performed in traditional clinical pathology. Terminology and definitions in this review are used according to current conventions in AI development. Figure 1 compares phases of test development, implementation, and monitoring in traditional veterinary diagnostic laboratory medicine and the development of an AI. The process of pre‐clinical evaluation (analytical method validation) in traditional clinical pathology parallels what is generally referred to as the model development (technical validation) and in silico testing phases of AI development. AI development is ideally fully transparent. Unfortunately, market competition disincentivizes transparency because it may mean that companies developing AI for commercial diagnostic or clinical use view AI development and function as proprietary.^18^

**FIGURE 2:**
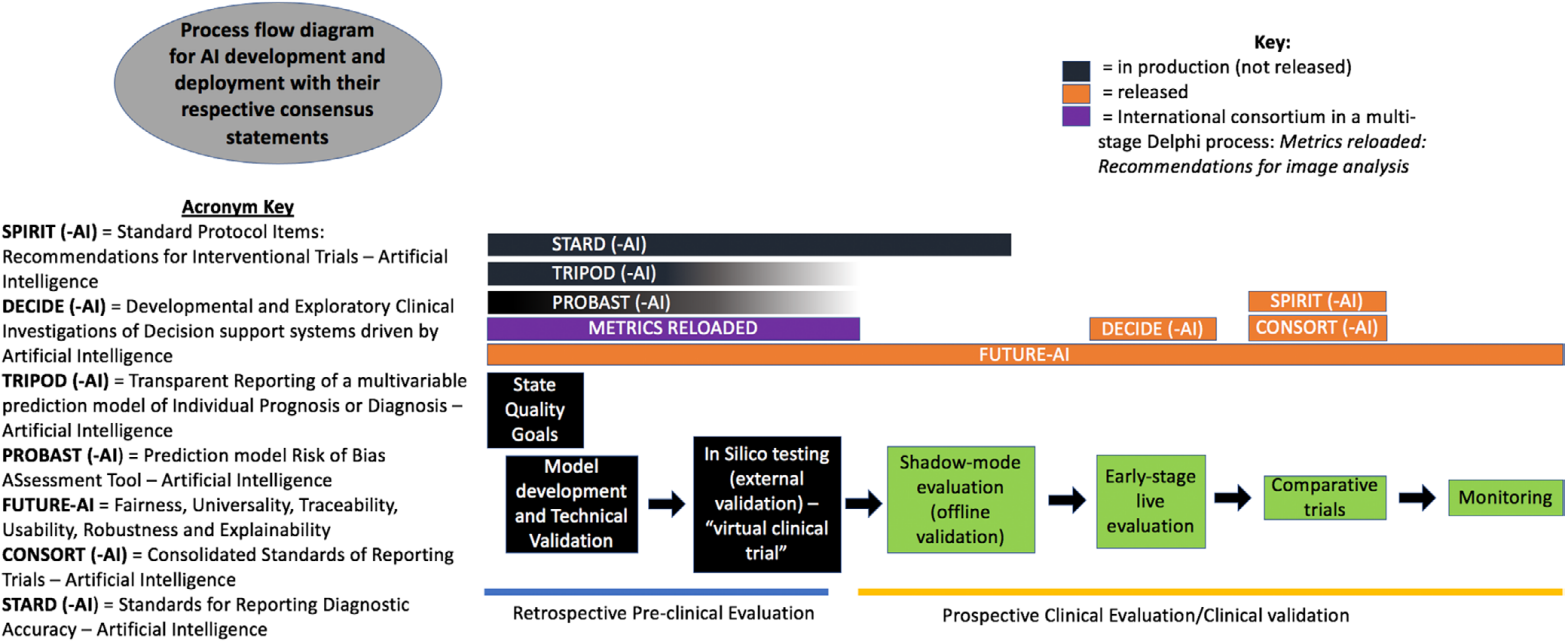
Given the rapid expansion of artificial intelligence with minimal guidelines on best practices for development and reporting, several consensus statements have been developed or are in development to address this concern and allow for a more transparent developmental and deployment process. This diagram introduces the various consensus statements and the area of development in which they are meant to be implemented. Some of the consensus statements are still in development. Therefore, a color key is provided that outlines which statements have been released or are currently in production. The stages of development and deployment in the schematic are adapted from Figure 1 and Table 1 of the Developmental and Exploratory Clinical Investigations of Decision support systems driving by Artificial Intelligence (DECIDE‐AI) guidelines^19^ and Figure 2 of the DECIDE‐AI: A new reporting guideline and its relevance to artificial intelligence studies in radiology paper.^20^

Several consensus statements are published or in preparation that describe best reporting practices for the different stages of AI development. Proposed development stages of AI intended for medical use are (1) model development, (2) in silico testing, (3) shadow‐mode evaluation (off‐line validation), (4) early‐stage live evaluation, (5) comparative trials, and (6) monitoring.^19^ Figure 2 is adapted from the DECIDE‐AI guidelines and presents a graphical overview of AI development stages together with the names of relevant consensus statements.^19^, ^20^ Model development and in silico testing are pre‐clinical stages. Other consensus statements targeting the preclinical stages of AI development include STARD (‐AI), TRIPOD (‐AI), and PROBAST (‐AI). These consensus statements remain in production and have not yet been published.^21^, ^22^ The FUTURE‐AI international guidelines provide recommendations that span all developmental stages of AI, including pre‐clinical development.

**FIGURE 3:**
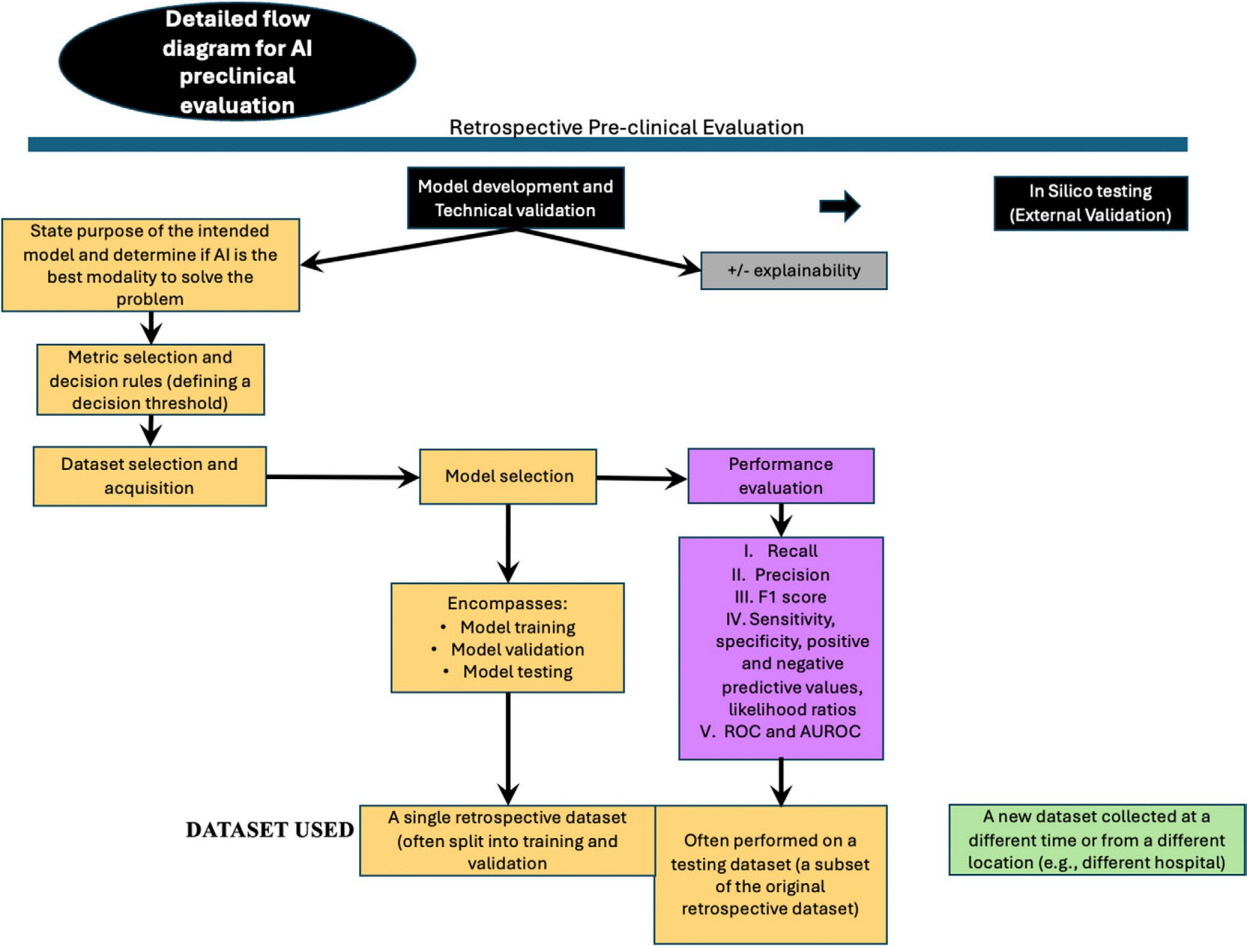
A comprehensive overview of the requirements for model development and technical validation of an artificial intelligence system (AI). This figure is adapted from the FUTURE‐AI and DECIDE‐AI guidelines.^20^, ^32^, ^49^

This section focuses on pre‐clinical AI development and presents considerations for (1) defining the problem and intended use of the AI, (2) determining if the AI can solve the problem to be evaluated, (3) selecting the appropriate metric for evaluation and defining a decision rule and threshold, (4) defining data acquisition and criteria, (5) selecting an AI model, and (6) evaluating AI model performance. Figure 3 outlines core considerations and topics that must be addressed to achieve successful preclinical production and evaluation of an AI. This review includes model development and technical validations. A companion paper will discuss in silico testing (external validation).^23^

### 2.1 Identifying AI deployment requirements

The first two steps of model development are (1) defining the problem and intended use of the AI and (2) determining if the AI can solve the problem to be evaluated. AI development requires a multi‐disciplinary approach, for example, obtaining input from computer scientists, statisticians, systems safety engineers, and medical professionals. In addition to carefully defining the problem to which the AI will be applied, other important considerations include the AI deployment environment (data collection, technological requirements, and infrastructure to implement and maintain the AI) and interpretability requirements (ie, to what degree the user should understand AI outputs).^24^ Failure to define the problem or identify deployment requirements can lead to the production of an AI that fails to solve the given problem or cannot be deployed in the intended environment because it does not meet the necessary criteria (eg, lack of interpretability, lack of explainability).

Various AI have different data requirements and, therefore, different computational and hardware needs. Some AI (eg, deep learning models) require large datasets to train for usable accuracy. Not only are many images/studies required, but each image or study often demands substantial storage space. For example, models that use whole slide images (WSI) require 2–3 gigabytes of storage per digital slide.^25^ Additionally, the computational power needed to run these models is considerable, often necessitating the purchase of newer computer systems capable of running these algorithms. Before hospitals can implement AI, they must have the technological (hardware/software) resources, policymakers, and operational experts capable of implementing, maintaining, troubleshooting, and validating these systems.^17^, ^26^ A potential benefit of AI is a reduction in healthcare costs; however, few studies have examined this in detail.^17^ Studies that show AI can reduce healthcare costs do not consider the cost of implementation and maintenance of AI.^27^ An article reviewing the cost of AI in 2021 cited variable implementation costs from $20 000 to $1 000 000 but was not specific to medicine and did not include the cost of system maintenance.^28^ Some authors posit that the resources required for an AI quality management system will be inhibitory without an industry partner or commercial support.^15^

In addition to considering the AI deployment environment, medical experts must be trained to use AI appropriately, including drawing accurate conclusions from the output.^26^ Interpretability is the ability of a person to understand or make sense of AI output.^29^ An example of interpretability may be an AI that detects lymphoma vs non‐lymphoma in canine patients based on cytology samples that give an output of 0.9. An output close to 1 means lymphoma, and an output closer to 0 indicates non‐lymphoma. A pathologist may look at the AI output, patient signalment, history, and physical exam findings and say, “This dog has lymphoma” (the interpretation). To make an accurate diagnosis, the pathologist optimally understands not only how the AI generates the 0.9 value (referred to as *the explainability of the AI, discussed further below*) but can also make sense of that output in light of other clinical information (good *interpretability* of AI output).

Explainability describes the process or rules an AI uses to achieve an output.^29^, ^30^ Explainability is considered a way for the AI model to gain the trust of medical professionals when the system is implemented and is often abbreviated as “XAI.” The National Institute of Standards and Technology (NIST) defines trustworthy AI as demonstrating the following characteristics: valid and reliable, safe, secure and resilient, accountable and transparent, explainable and interpretable, privacy‐enhanced and fair (managing bias that may be harmful).^31^ Given the close relationship between trust and explainability, some argue that all AI intended for medical use must be explainable. A salience heat map is one method of adding explainability to an AI. Based on different colors, a salience heat map highlights what parts (pixels) of an image are used to generate the output supporting a clinical decision. In the above lymphoma scenario, the AI can be considered explainable if, for example, the pathologist can review a salience heat map to understand the generation of the 0.9 output value interpreted as “lymphoma.” The AI output of 0.9 is more credible if the saliency map emphasizes large, immature lymphocytes vs some other feature of the slide (eg, an artifact like free nuclei or something in the background). Explainability does not necessarily equal AI credibility, but it can improve trust in an AI by helping a pathologist judge if AI output seems accurate and applicable to the disease in question.

Others firmly believe that rather than emphasizing explainability, trust in an AI can be gained from rigorous testing and prospective studies showing a positive impact of the AI on the clinical outcome.^24^ Given these various opinions, it is recommended that medical professionals help define how explainable a given AI should be for its intended purpose.^24^, ^32^ The FUTURE‐AI guidelines suggest that both interpretability and explainability are needed for AI to be used in medicine.^32^ The FUTURE‐AI guidelines do not mandate that AI in healthcare needs to be explainable, but they recommend that explainability requirements be discussed before the model is developed.^32^ The European Union and its regulatory bodies have implemented a “right to explanation” that calls for greater algorithmic accountability in AI development. The General Data Protection Regulation (GDPR), with provisional clauses from Rictals and Working party guidelines, prohibits companies from making decisions that “significantly impact” an individual solely based on AI‐algorithmic tools.^33^, ^34^ It should be noted that there could be potential legal ramifications if an AI is not explainable.

In summary, a holistic approach to defining the intended purpose and deployment setting of an AI that involves all professionals (eg, end users and engineers) in producing the AI from the beginning is key to the model's success. This approach helps ensure that the AI production accounts for the resources required to create, implement, and maintain it in its intended setting. It also allows for intended use and quality assurance to be discussed early in development.

### 2.2 Metric selection and decision rules

Before the start of AI development, metrics that appropriately evaluate the success of an AI should be selected. A metric is any measurement used to quantify and validate the performance of an AI.^35^ Therefore, metric selection describes choosing the appropriate validation metrics. For instance, if the AI system is designed to classify a patient as having large cell lymphoma or non‐lymphoma, the accuracy of the model output, sensitivity, specificity, and AUROC may be chosen as appropriate metrics. These metrics will then be used throughout the development and evaluation process of the AI to determine its success in completing the task.

Metrics are commonly based on some form of the confusion matrix when the AI is used to classify or label a whole image (eg, the lymphoma, non‐lymphoma example). This matrix allows for the extraction or visualization of standard metrics used for performance evaluation.^36^ The confusion matrix often comprises sensitivity, specificity, negative and positive predictive value, or accuracy.^35^

Poor metrics selection is suspected to be a significant cause of AI failure when deployed in clinical settings. Risks of poor metric selection include failing to correctly identify an AI that will answer the question being asked, failure of the system to perform well in the intended environment (eg, clinical setting), or misleading claims on the success of an AI^37^, ^38^ Depending on the input and goals of the AI, various ways are available to create decision rules for validation. Meier‐Hein et al^35^ provide a detailed explanation of the process to assist researchers in selecting appropriate metrics for image analysis‐based AI. A decision rule is a rule that transforms a continuous AI output into a discrete decision class by assigning a cutoff or decision threshold to a classification category. Meier‐Hein et al^35^ describe four different decision rules that can be applied and validated: (1) target‐value based—a specific target value must be achieved, for example, specificity = 80%, (2) optimization based—optimization of a specific metric, for example, optimize sensitivity, (3) Argmax‐based—cutoff is based on picking the class with the highest class score, (4) cost–benefit‐based—risk assessment based, for example, cost of 20 un‐necessary invasive procedures is 4 detected malignant lesion and (5) no decision ruled applied—an algorithm is not validated using a specific decision rule and only the results of the multi‐threshold metric is used. For example, if an AI was being made to differentiate lymphoma and non‐lymphoma using cytology samples and the user wanted it to perform as well as a clinical pathologist, then a target‐based method may be chosen. The literature suggests clinical pathologists have a ~ 93% sensitivity in diagnosing lymphoma.^39^ Using a target‐based decision rule validation, the researchers may set a decision‐rule validation threshold of sensitivity = 94%, with the goal that the model performs better than a clinical pathologist.

Decision rules can be chosen during the validation phase (decision rule‐based validation) or later in model development (ie, after the validation phase, referred to as decision rule‐agnostic validation).^35^ Some specialized fields of AI require decision rule‐based validation, but more recently, the literature suggests that decision rule‐agnostic validation is recommended for clinical AI.^35^ This is because the decision rule(s) may need to be changed if the AI is deployed in an environment that differs from the validation environment. Decision rule changes may be necessary due to differences in disease prevalence and clinical application of the AI output (cost–benefit to the patient may vary across hospitals).^35^ To ensure the success of an AI for its intended use, all contributors to the production of an AI should decide whether decision rule‐based validation or decision rule‐agnostic validation is the best choice to achieve a given goal.

### 2.3 Defining data acquisition and criteria

The dataset chosen to train an AI is critical to success.^40^ Therefore, the acquisition processes for AI training data should be carefully documented to ensure a safe, trustworthy, fair, and generalizable AI. Examples of bias that can impact the success of AI in veterinary medicine are given in the subsequent paragraphs.

The data acquisition processes should be defined before the AI is developed. Both inclusion and exclusion criteria of the training dataset should be predetermined. Clearly stating data sources will help identify potential sources of bias within the dataset and allow for early mitigation plans to address bias. Additionally, the researchers should ensure that the training dataset selected represents data the AI will encounter when implemented in the clinical environment (ie, the training dataset will ensure the AI is appropriate for the desired context of use).

#### 2.3.1 Data distribution variations and bias

When implementing an AI, significant concerns are sources of bias, overfitting, and underfitting. Problems can occur if models are trained with unevenly distributed data. For example, when creating an AI to detect lymphoma vs non‐lymphoma based on cytology samples, if all lymphoma cases are imaged at hospital A and all non‐lymphoma cases are imaged at hospital B, then the imaging equipment is a source of possible bias. In such circumstances, the AI might detect differences in the imaging modality (ie, image quality or characteristics) or staining technique rather than detecting genuine predictive features of negative and positive patient data.^26^ Another example of bias introduced by patient location is an AI trying to predict the length of hospital stays in dogs diagnosed with parvo. A potential source of bias in this model may be that hospital A (high‐income area) has more extended hospitalization stays for parvo cases than hospital B (low‐income area) because location A owners can afford to keep patients in the hospital longer. If the model is trained using data only from hospital A (or only from hospital B), bias is introduced. Another potential source of bias from this example is that most parvo‐positive dogs are puppies, so if the model is trained using data from young dogs, the length of stay in the hospital may not be accurate when applied to adult dogs. The model becomes biased by patient age.

#### 2.3.2 Data collection and labeling variations

Specimen processing, data collection, and storage vary considerably across institutions. Using anatomic pathology as an example, there is a lack of standardization for tissue sectioning (tissue thickness), staining protocols, and image collection (different cameras, computer settings, whole slide scanner systems, and magnifications). Such differences mean that applying AI universally across various hospital environments may not be possible.

Additionally, digital datasets may vary in the amount of labeled (meta) data,^26^ variations in opinions between specialists (ie, inter‐observer variation), and a lack of standardized terminology for the labels assigned.^41^ The impact of inter‐observer variation will differ for different fields of medicine and tasks in each sub‐specialty. For example, the inter‐observer agreement can vary from poor to good in veterinary pathology, depending on the diagnosis.^42^, ^43^ Therefore, data labeled by multiple pathologists with poor inter‐observer variation may produce inaccurate or biased AI. In contrast, if a single pathologist labels all the data, then there may be intra‐observer variation introduced into the system.

### 2.4 AI model selection

Model selection is a significant part of AI preclinical development. *Selection* is the process of training and evaluating different AI models to determine which is the best for an intended use. One of the most common model selection methods is using the 3‐way holdout method with a k‐fold cross‐validation. Here, selection components include (1) model training, (2) model validation, and (3) model evaluation.^44^ One of the most significant differences between clinical pathology and AI is the use of word *validation*. Model validation in AI evaluates a set of hyperparameters (external variables) to determine the best model for a given task.^45^ Hyperparameters (external variables) are any parameters the researcher specifies before training the model. In contrast, the researcher does not change internal variables (the algorithm learns them) such as [weights and bias]. Examples of hyperparameters include learning rate, batch size, number of layers in the network, and dropout. An example of hyperparameters and parameters when building a truck might be that hyperparameters include if there are heated seats, number of doors, size of the engine, and parameters might be that seatbelts, a steering wheel, and four tires are always present (never changed). For more information and examples of hyperparameters, see Table 1. In contrast to traditional clinical pathology, where “method validation” means demonstrating that an assay is acceptable for its intended clinical use, model validation in AI development^45^ commonly refers to determining which hyperparameters will be used to optimize model performance.^45^ The use of the term validation in AI development has been inconsistent, and procedures variably described as external validation, offline validation, and clinical validation appear to set a goal of determining, through rigorous testing, the acceptability of the AI for its intended use.^19^, ^40^

**TABLE 1:** Parameters and hyperparameters are important components of an AIS. This table defines terms and gives examples of each.

	Parameter		Hyperparameter	
Definition	Distinguishing features or variables the AI learns during training and then uses to make its predictions. These variable are not typically determined by the designer of the AI. Dependent on the dataset used to train the model.		A set of parameters that are set by the researcher before the model is trained. Independent from the dataset used to train the model.	
	Term	Definition	Term	Definition
Examples	Weights	Learnable parameter that help convey how important a feature is to make a prediction and the relationship between a feature and the target value	Learning rate	Controls how much the model updates (changes) its parameters in response to its estimated error when compared to the ground truth
	Biases	Biases are a constant, associated with each neuron. Biases allow the neuron to activate even when sum of the inputs is not enough to activate a neuron on its own. Their roll is to introduce flexibility and adaptability to the model	Number of epochs	The number of times the model will iterate (cycle) through the entire training dataset to reach the best performance. Each epoch contains within it a defined number of batches (see batch size)
			Loss Function	Mathematical function that quantifies the degree of error between the model's prediction and the ground truth
			Batch size	The number of datapoints (eg images) to pass through the model at a time before the internal parameters are updated.
			Number of hidden layers	Hidden layers extract the important features from the input. The hidden layer is between the input layer (ie, where the data is put into the model) and the output layer (ie, the information we receive from the AI)

#### 2.4.1 Model selection with the 3‐way holdout method

**FIGURE 4:**
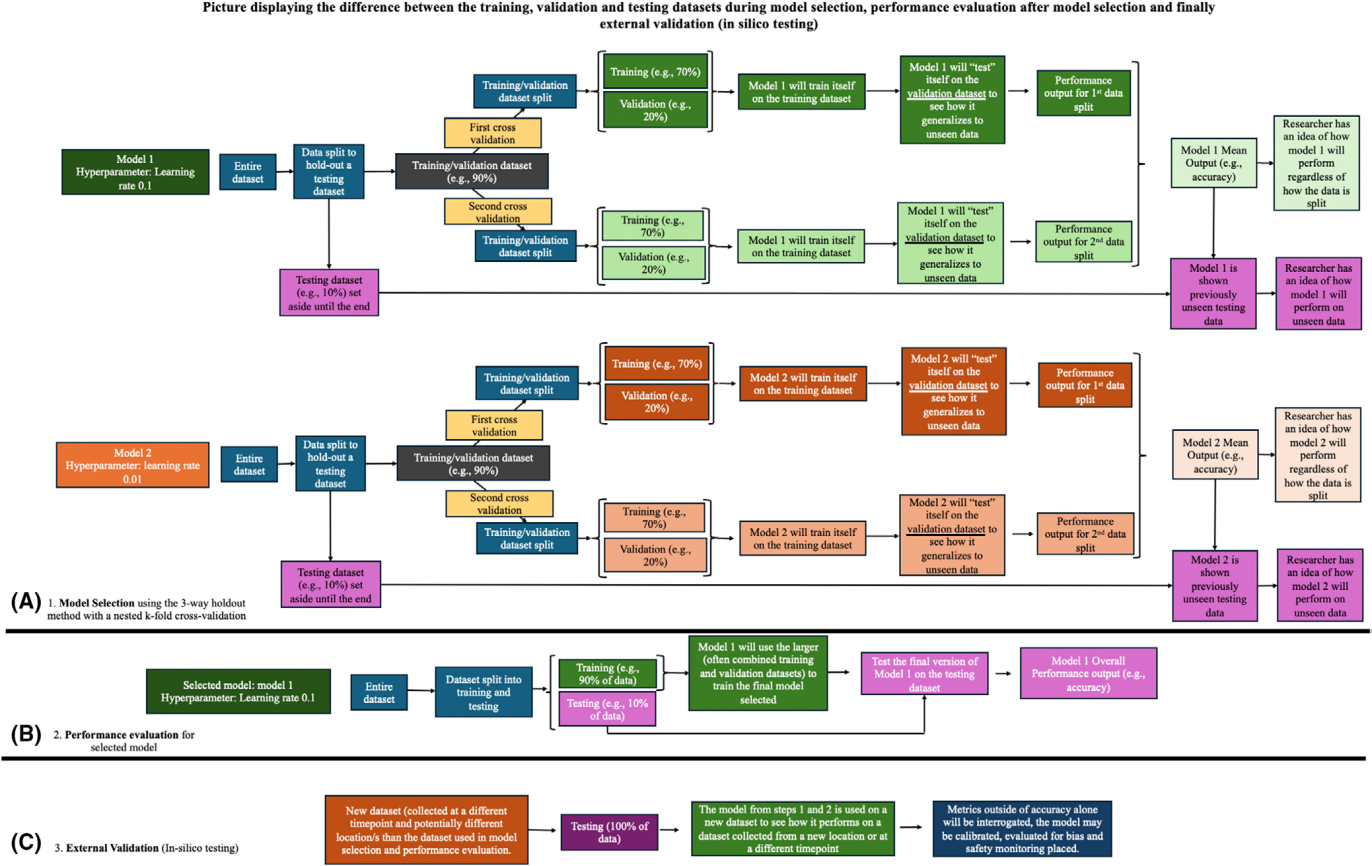
This figure, with parts A, B, and C, visualizes the differences between model selection, performance evaluation, and external validation, respectively. (A) Model selection is the process of determining which model (or hyperparameters) produces the best performance. One way to perform this is with the 3‐way holdout method and cross‐validation. To perform a 3‐way holdout, the dataset is split into two piles: (1) training/validation and (2) testing dataset. The training/validation dataset is further split into a (1) training dataset and a (2) validation dataset. The model is trained on the training dataset and then evaluated on the validation dataset. To ensure that the way the dataset is randomly partitioned is not a factor in the model accuracy, a k‐fold cross‐validation is performed. Here, the data is repeatedly split so that each datapoint has the chance to be in the training and validation dataset, respectively. The accuracy of each data split (ie, cross‐validation) is averaged to give an idea of how well the model will perform. Because the data is reused during this process, a small subset called the “testing” dataset is set aside. The goal of the testing dataset is to determine how well the model will generalize to previously unseen data. (B) After the best model is selected, then this model will undergo an additional performance evaluation. Here, the entire dataset is split into two (instead of three) piles: (1) training and (2) testing. The idea behind performance evaluation is that ML models perform better the more data they are shown. Therefore, by splitting the dataset into only two piles, a greater portion of the data can be used to train the model and a final model performance evaluation is determined. (C) After performance evaluation, the model can go through an external validation process. Here, the final model from the previous steps is shown a new dataset that was collected at a different time or from a different source (eg, different hospital) than the original data.

One of the most well‐known procedures for AI model selection is the 3‐way holdout method. Here, a large dataset is divided randomly into separate sets, one each for training, validation, and testing.^44^ The terms *training*, *validation*, and *testing* are used here according to the convention of AI literature. Many different AI models with differing hyperparameters are made as part of AI development. Candidate models are then trained using the training dataset. Training allows candidate models to learn the internal variable values of the model that are best at making accurate predictions for any given task. Next, to evaluate the internal variable values' success in correctly classifying images, the models are shown the validation dataset. Each model's performance output (eg, accuracy) is then compared. Lastly, each model is shown the testing dataset, and the model's performance outputs are compared. Output based on the testing dataset is considered the final performance of the model.^44^ The model with the best performance is selected and interpreted to have the best hyperparameter settings. A visual representation of this process using AI is presented in Figure 4A.

#### 2.4.2 An applied example of the 3‐way holdout method using accuracy as the performance metric

Returning to the example of researchers creating an AI that can differentiate cytology images of large‐cell lymphoma versus non‐lymphoma, consider how we decide which is the best model based on accuracy. The dataset for model selection consists of 100 images of large‐cell lymphoma (class 1) and 100 non‐lymphoma (class 2). To perform model selection using the 3‐way holdout method, the 200 images are randomly divided into balanced training, validation, and testing sets. A common way to split the data is 70% training, 20% validation, and 10% testing.^46^ It is important to note that a balanced distribution of each diagnosis class (large‐cell lymphoma and non‐lymphoma) is maintained within each subset.

**FIGURE 5:**
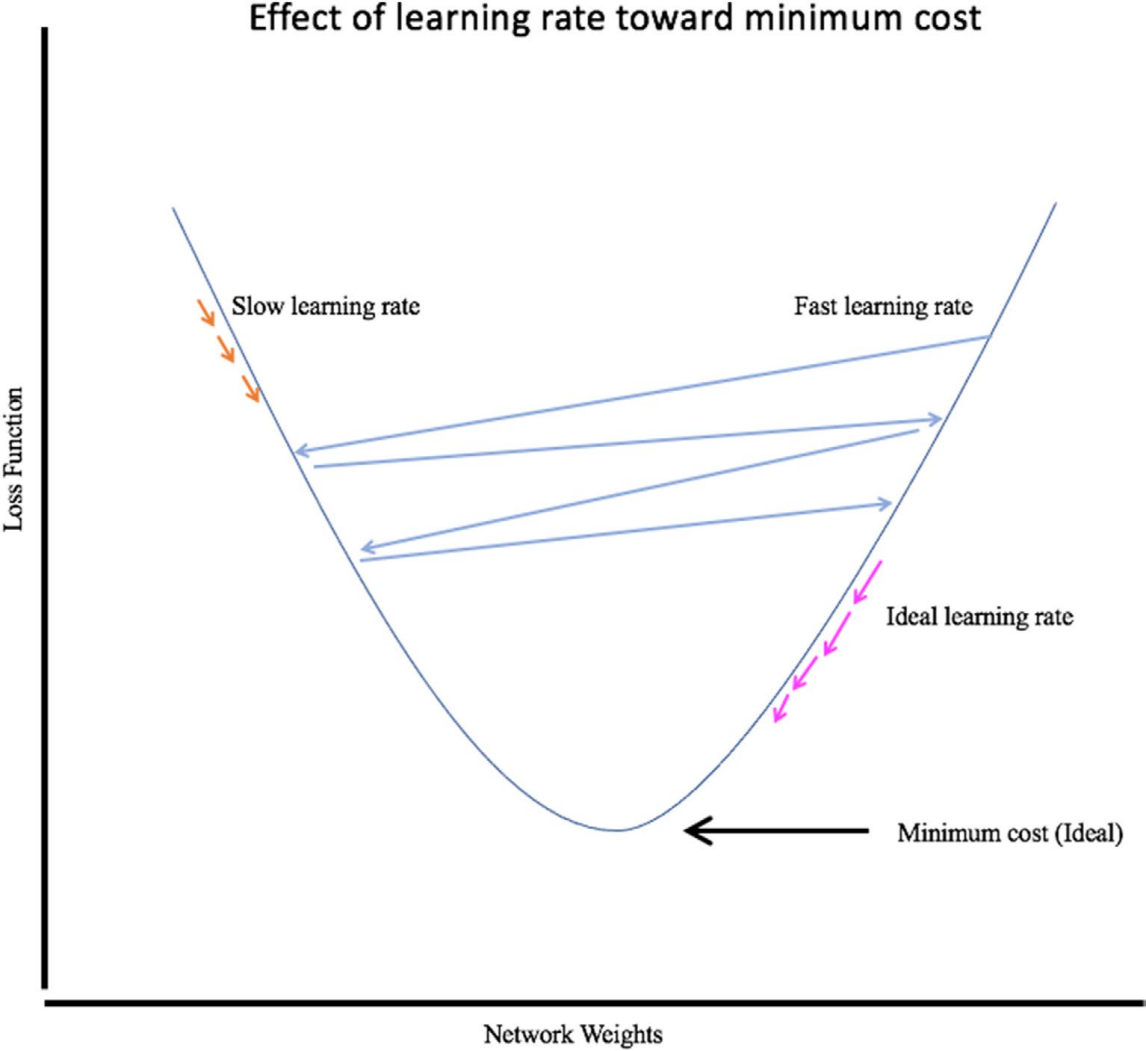
Depiction of the effects of different learning rates. If the learning rate is too slow, the model will take too long to train. If the learning rate is too fast, it will oscillate too much to effectively find the best model that minimizes the loss function. The loss function is the difference between the true value and the predicted value. If a model frequently labels images of large‐cell lymphoma as non‐lymphoma, the model will have a high‐loss function. If the model labels the lymphoma images as lymphoma (correct prediction), then the model has a low‐loss function (ideal). Minimization of the loss function in an AI is commonly referred to as the gradient descent.

Next, several different hyperparameters are chosen for each model before model training begins. The learning rate is a common pre‐determined hyperparameter. (Note that the term *rate* is a misnomer, as there is no time component.) The learning rate is a unitless value that determines how much change the model will make each time it goes through the training data as it moves closer to the optimal solution (see Figure 5). For example, the researcher may set Model 1 to have a learning rate of 0.1 and Model 2 to have a learning rate of 0.01. These models are then shown the training dataset, where they learn the predictive parameters that lead to a correct output classification (eg, large‐cell lymphoma or non‐lymphoma). Predictive internal learned parameters differentiating large‐cell lymphoma from non‐lymphoma using light microscopy may include lymphocyte size, chromatin pattern, and homogeneity or heterogeneity of the lymphocyte population. These AI‐learned internal predictive parameters may also include features imperceptible to the human eye, for example, the number of pixels that contain the color blue.

The researcher must determine which candidate model performs best after training by showing each validation dataset. In this example, the researchers use accuracy as the metric to choose the best candidate model. The best‐performing model is selected by looking at each model's validation dataset output (accuracy). Lastly, the newly trained model is shown a testing dataset to estimate the overall performance of the chosen model on an unseen dataset.

#### 2.4.3 Model selection using 3‐way holdout and cross‐validation

**FIGURE 6:**
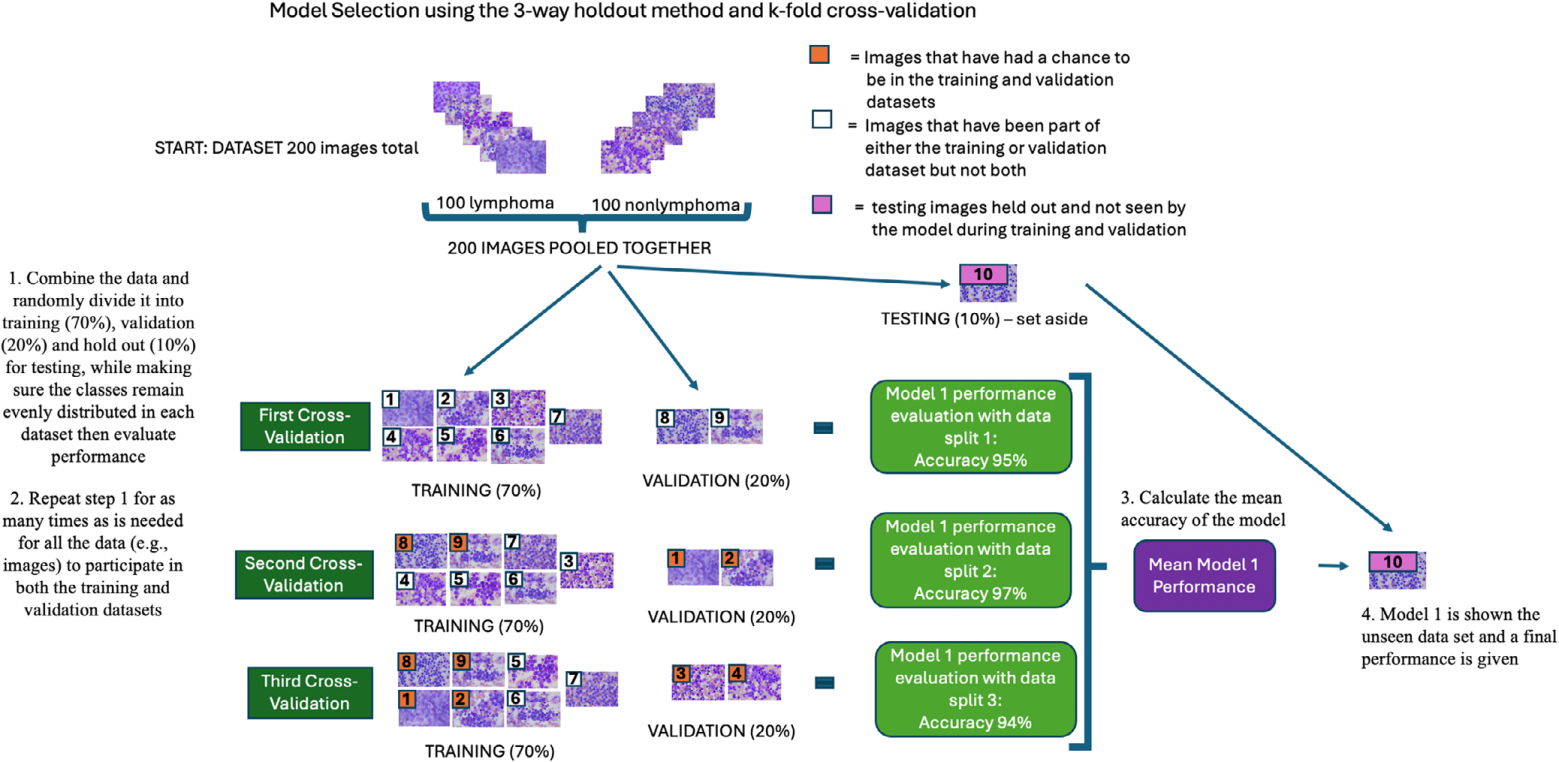
This diagram provides a visual representation of k‐fold cross‐validation. The goal of cross‐validation is to allow each part of the data to be in the training and validation datasets, respectively. K‐fold cross‐validation can be used during model selection and model evaluation. In this example, an attempt is being made to use lymph node cytology images from canine patients to differentiate large‐cell lymphoma from non‐lymphoma (lymphoid hyperplasia). This figure is adapted from Figure 16 of the Model Evaluation, Model Selection, and Algorithm Selection in Machine Learning paper.^44^

How data are partitioned into training, validation, and testing subsets during model selection can affect which parameters each candidate model determines are the most predictive and the model's accuracy. A k‐fold cross‐validation procedure can be employed to minimize the effects of data partitioning bias on model performance.^44^ The main feature of k‐fold cross‐validation is that each data subset can be used for training and validation in a series of training and validation cycles. After each training and validation cycle, the dataset is split again into training and validation subsets, but in a different order, so each input (datapoint) is ultimately used for both training and validation. During each training and validation cycle, performance output estimates for each candidate model are calculated, and the best model is selected. K‐cross‐fold validation can be used for model selection and model performance evaluation. Due to the extra computational power required to perform cross‐validation, it is generally reserved for small datasets.^44^ Figure 4A and Figure 6 provide a visual representation of cross‐validation.

Confidence intervals can be generated for a large dataset using normal approximation. For smaller datasets, cross‐validation with bootstrap methods may be used to determine a confidence interval (uncertainty) surrounding the output.^44^

### 2.5 Model performance

A final performance estimation is performed once a candidate AI model is selected. Because AI works best when trained on a large dataset, the researchers often pool the training and validation datasets to create a final, larger training dataset for the selected best model (ie, performance evaluation step). Sometimes, the model/performance evaluation step of model selection is confused with the overall performance evaluation. These are separate and distinct processes. The model evaluation is part of the model selection process and is performed separately using the training and validation datasets. In contrast, the end performance evaluation uses a greater portion of the dataset to train the final model and determine a final overall performance (i.e., the training and validation datasets are pooled to create a single, larger training dataset.)^44^ See Figure 4 for a visual representation of the relationship between model selection, performance evaluations, and in‐silico testing.

As mentioned in section 2.2, an important part of performance evaluation using the testing dataset is determining the metrics and decision rules used to estimate model performance. Performance estimate metrics should be defined before the start of the study. Determining the metrics best suited for performance estimation will depend on the study's goal and the type of AI used. Inappropriate metric selection leading to over‐exaggeration of the model performance or improper performance evaluation given a specified question are among image analysis studies' central performance evaluation problems.^37^ For example, an AI that classifies an entire image as lymphoma or non‐lymphoma (image‐level classification) vs an AI that partitions/labels an image into different regions (such as a single image labeled with areas of lymphocytes, background, red blood cells, known as semantic segmentation) will require different metrics. Continuing this example, semantic segmentation requires metrics that evaluate the overlap of the partitioning labels and the label boundaries (e.g., how much pixel overlap exists between what the AI called ‘lymphocytes’ and what it called ‘background’), whereas image‐level classification does not. To minimize these limitations, Maier‐Hein et al, released a paper in 2024 entitled *Metrics reloaded: Pitfalls and recommendations for image analysis validation*.^35^ This paper provides a comprehensive guide to metric selection for performance evaluation, specifically for image analysis studies. Given that the responsibility of quality assurance falls to pathologists, it is critically important that they understand best practices for AI metric performance, not only so they can assess if appropriate metrics were used to evaluate an AI when reading the literature but also so they can make best practice recommendations when consulted during production of AI for use in pathology.^37^ A full review of metrics used to evaluate AI performance is beyond the scope of this review, and interested readers should consult other resources.^35^ A brief summary of AI performance evaluation relevant to veterinary pathology is provided below.

Maier‐Hein et al divide image studies into four broad categories: image‐level classification (an entire image is classified with a label); semantic segmentation (pixels are classified); instance segmentation (classification at object/pixel level); or object detection (classification at an object level).^35^ Determining what category of image study is needed will inform metric selection recommendations. For example, image‐level classification (eg, assigning a cytology image as large‐cell lymphoma or non‐lymphoma) first requires the selection of a multi‐class counting metric/(s). Examples of metrics that fall into this category include but are not limited to accuracy, balanced accuracy (BA), Mathews Correlation Coefficient (MCC), sensitivity, specificity, and positive/negative predictive values. The multi‐class counting metric gives a holistic overview of the model performance, considering all classes (in our example, large‐cell lymphoma and non‐lymphoma). Multi‐class counting metrics do not provide an assessment of each class individually. Therefore, a multi‐threshold metric such as the area under the receiver operating characteristic curve (AUROC) or average precision (AP) is recommended to assess individual classifications. Lastly, per‐class counting metrics such as F_ß_ score, sensitivity, specificity, positive likelihood ratio, and positive predictive value can be performed.^37^ Notice that many of these metrics are commonly used in diagnostic performance studies, which in traditional clinical pathology, follow analytical method validation.

An important concept, the ‘AI chasm,’ refers to the discrepancy between the model performance of AI system in a research setting and real‐world application. Even if an AI has high model performance in the developmental environment, the AI may not be accurate or generalizable in real‐world diagnostic settings.^15^, ^16^, ^17^ Regarding AI intended for medical applications, this chasm is due, in large part, to differences in methodology between computer scientists and medical professionals.^47^ To begin to fill this gap, medical professionals and computer scientists need to understand these differences so they can be addressed. Prospective studies are required to better understand the relationship between the accuracy metric and clinical efficacy.^15^, ^16^

## 3 CONCLUSIONS

AI is a diagnostic tool receiving considerable interest in medicine. AI technology can process and analyze vast amounts of data at speeds and scales unachievable by individual human clinicians. This capability can potentially improve the diagnosis of complex diseases, the interpretation of imaging studies, and the prediction of the onset of certain conditions or patient outcomes. However, as with any technological advancement, AI has its challenges.

To ensure that veterinary medicine maintains high standards of care, it is essential to develop AI systems using a holistic approach that combines end‐user expertise with computational and risk assessment experts. This process should be followed from the developmental phase of the system to its use in the clinic. AI must undergo the appropriate developmental, validation, and verification phases like all diagnostic tools. These phases should be performed with transparency and following the proper guidelines. Additional research showing the relationship between clinical efficacy and accuracy of AI is needed before their widespread implementation.

One of the primary concerns has been the ethical implications of AI in clinical settings. The questions of data privacy, consent, and the potential for bias within AI algorithms require ongoing attention. Ensuring that AI systems are developed and deployed responsibly is crucial to maintaining trust in this technology.

The success of AI applications in veterinary medicine starts with a holistic approach from the beginning, involving all stakeholders in the developmental process to ensure that AI will fulfill its purpose. This will require training, education, and a shift in the traditional approach to veterinary care. When an AI is used in a clinical setting, appropriate resources must be provided to provide continuous quality assurance. Lastly, a better understanding of the cost–benefit and cost of implementing and maintaining these systems is required.

Part II of this review covers the AI development step that follows technical validation and external validation (in silico testing). Topics related to assuring the quality of an AI, such as evaluation for bias, uncertainty, run‐time monitors (safety), generalizability assessment, robustness, repeatability, and the effects of calibration, will be discussed.

## CONFLICT OF INTEREST STATEMENT

The authors have indicated that they have no affiliations or financial involvement with any organization or entity with a financial interest in, or in financial competition with, the subject matter or materials discussed in this article.

## APPENDIX A

### A.1

1. Artificial Intelligence (AI): Computers performing tasks that usually require human intelligence, like recognizing patterns or solving problems.
2. Neural Networks: A series of algorithms that mimic the human brain's operations to recognize relationships in a data set.
3. Deep Learning Models: A type of AI that learns and makes decisions by analyzing large amounts of data, similar to how humans learn from experience
4. In Silico Testing: Testing AI models using computer simulations instead of real‐world experiments.
5. Algorithm: A set of rules or instructions given to an AI program to help it learn and make decisions.
6. Data Acquisition: The process of collecting data that will be used to train and test AI systems.
7. Model Development: Creating and designing an AI model includes selecting the right approach and setting performance goals.
8. Bias: In AI, bias refers to the tendency of an algorithm to favor certain outcomes based on its design or the data it is trained on.
9. Calibration: Adjusting AI predictions to make them more accurate and reliable in real‐world applications.
10. Generalizability: The ability of an AI system to apply what it has learned from specific data sets to new, unseen data.
11. Robustness: The strength of an AI system to maintain performance even when faced with challenging or unexpected conditions.
12. Uncertainty: The confidence level in an AI system's predictions. High uncertainty means the system is less sure about its predictions.
13. Explainability: The ability of an AI system to clearly explain how it arrived at its decisions or predictions.
14. Run‐time Monitoring: The process of continuously checking an AI system as it operates to ensure it is working correctly and safely.
15. Variance: How the performance of an AI system can be impacted by changes in the data used to train it.
16. Overfitting: When a model performs well on the training dataset but poorly on a new, unseen dataset.
17. Underfitting: When a model performs poorly on the training dataset and new, unseen dataset because it cannot capture the relationship between the input and output.
18. Underspecification: When several different models perform well on the training dataset, but when deployed, their performance decreases due to differences in the training and deployment environments.
19. Artificial intelligence model: a system trained to recognize specific patterns from a provided dataset
20. External dataset: A dataset derived from a different source than the internal dataset.
21. Internal Dataset: The internal dataset is often derived from information collected from within a specific organization (e.g., hospital) and is often used to train an artificial intelligence model
22. External validation: Evaluation of a model using a dataset that was collected separately from the training (internal) dataset
23. Stress test: any test used to evaluate the stability and robustness of a model
24. Model: A program that detects a specific pattern learned from training data
25. Parameter: Distinguishing features or variables the AI learns during training and then uses to make its predictions.
26. Hyperparameter: A set of parameters that the researcher selects before the model is trained.
27. Cost function: The average of the loss functions from the training dataset
28. Loss function: Difference between the actual and predicted values of a machine learning model on a single instance (e.g., single datapoint)
